# The free β-subunit of human chorionic gonadotropin as a prognostic factor in renal cell carcinoma

**DOI:** 10.1038/sj.bjc.6600050

**Published:** 2002-01-21

**Authors:** K Hotakainen, B Ljungberg, A Paju, T Rasmuson, H Alfthan, U-H Stenman

**Affiliations:** Department of Clinical Chemistry, Helsinki University Central Hospital, Biomedicum Helsinki, Rm A418a Haartmaninkatu 8, FIN-00029, Helsinki, Finland; Department of Urology and Andrology, Umeå University, S-901 85, Umeå, Sweden; Department of Oncology, Umeå University, University, S-901 85 Umeå, Sweden

**Keywords:** renal cell carcinoma, stage, grade, hCGβ, prognosis

## Abstract

The free β-subunit of human chorionic gonadotropin β is expressed in several nontrophoblastic tumours and this is usually associated with aggressive disease. Little is known about human chorionic gonadotropin β expression in renal cancer. We determined the pretreatment levels of human chorionic gonadotropin β in serum of patients with renal cell carcinoma, and studied whether elevated levels predicted the clinical outcome. Serum samples were collected before surgery from 177 patients with renal cell carcinoma and from 84 apparently healthy controls. Human chorionic gonadotropin β in serum was measured by a highly sensitive time-resolved immunofluorometric assay. The prognostic value of human chorionic gonadotropin β, and of usual clinical and pathological variables was analyzed by the Kaplan-Meier method, the log rank test and Cox multiple hazard regression. The serum concentrations of human chorionic gonadotropin β were increased in 23% of the renal cell carcinoma patients and they were significantly higher in patients with renal cell carcinoma than in controls (*P*<0.0001). The concentrations did not correlate with clinical stage and histopathological grade, but patients with increased human chorionic gonadotropin β levels had significantly shorter survival time than those with levels below the median (cut-off 1.2 pmol l^−1^, *P*=0.0029). In multivariate analysis human chorionic gonadotropin β, tumour stage and grade were independent prognostic variables. The serum concentration of human chorionic gonadotropin β is an independent prognostic variable in renal cell carcinoma. The preoperative value of human chorionic gonadotropin β in serum may be used to identify patents with increased risk of progressive disease.

*British Journal of Cancer* (2002) **86**, 185–189. DOI: 10.1038/sj/bjc/6600050 www.bjcancer.com

© 2002 The Cancer Research Campaign

## 

The incidence of urological malignancies such as prostate cancer, bladder cancer and kidney cancer has been steadily increasing in the Western world during the past decades (Parkin *et al*, 1999). Renal cell carcinoma (RCC) arising in the renal parenchyma is the most common malignancy of the kidney. Conventional renal cell carcinoma (CRCC) and papillary renal cell carcinoma account for 85–90% of the five distinct types of RCCs. The fairly indolent chromophobe renal cell carcinoma and the unclassified category account for approximately 5% each, while the rarest and most aggressive type is collecting duct carcinoma (Kovacs *et al*, 1997). Clinical stage and nuclear grade are the most important prognostic factors in RCC (Fuhrman *et al*, 1982), but within a given disease stage the outcome is highly variable, and other prognostic indicators are needed to identify patients with high risk of progressive disease (Golimbu *et al*, 1986). Cell proliferation markers, p53 mutations, growth factor expression, and intratumoural microvessel density have been investigated as prognostic indicators, with partly contradicting results (Gelb *et al*, 1997; Grignon *et al*, 1995). Among the serum markers studied, VEGF, interleukin-10, CA-125, and tumour-associated trypsin inhibitor (TATI) have been shown a prognostic value, but no specific markers are available (Grankvist *et al*, 1997; Jacobsen *et al*, 2000; Paju *et al*, 2001; Wittke *et al*, 1999).

Human chorionic gonadotropin (hCG) is a 38 kDa glycoprotein hormone consisting of two dissimilar subunits, α and β (hCGα and hCGβ) (Bahl *et al*, 1972). HCG is normally produced by trophoblasts during pregnancy and it is a very sensitive marker for trophoblastic tumours (Vaitukaitis and Ebersole, 1976). Expression of hCGβ is a fairly common phenomenon in several non-trophoblastic tumours (Alfthan *et al*, 1992a; Marcillac *et al*, 1992), and in transitional cell carcinoma of the bladder (Iles *et al*, 1989) and several other tumours this is associated with aggressive and therapy resistant disease. Tissue expression of hCGβ seems to have prognostic significance in carcinomas of the prostate (Sheaff *et al*, 1996), and hCGβ in serum has been found to be of prognostic value in ovarian cancer (Ind *et al*, 1997). The aim of this study was to evaluate the prognostic value of hCGβ in sera of patients with RCC in relation to established prognostic factors.

## MATERIALS AND METHODS

### Patients

Data for this retrospective analysis was collected from 177 patients with RCC from whom pretreatment serum samples were available. The patients underwent radical nephrectomy at the University Hospital of Umeå, Sweden between 1983 and 1995. The study included 111 men and 66 women with a mean age of 65 years (range 25–85) (Table 1

**Table 1:**
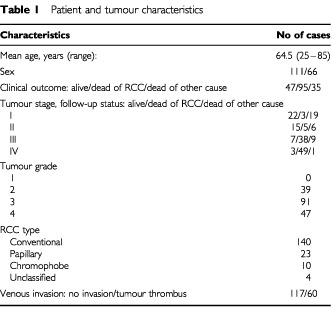
Patient and tumour characteristics

). Informed consent was obtained from all patients included in the study. Serum samples were collected before surgery and stored at −80°C until analysis. Staging procedures included physical examination, chest radiography, ultrasonography and computer tomography. Patients with skeletal symptoms or elevated serum alkaline phosphatase were assessed by bone scintigraphy and skeletal radiography. After nephrectomy, all patients were followed up with clinical and radiological examinations. At the last follow-up, 47 of 177 patients were alive with a median follow-up time of 124 months, (range 34–191 months). Among 130 patients that had died, 95 had died of RCC and 35 of other causes (Table 1). Sera from 84 apparently healthy volunteers was used as a reference group.

### Tumour staging

Tumour staging was performed according to 1997 TNM Stage classification (Sobin and Wittekind, 1997). Among the 177 patients, 70 patients had TNM stage I and II (pT1–pT2, N0, M0), 54 stage III (pT1–T3a–c, N0–N1, M0) and 53 were of stage IV (pT1–T4, N0–N3, M1). Nuclear grading was performed according to Skinner *et al* (1971) and DNA ploidy according to Ljungberg *et al* (1996).

### Serum determinations

HCGβ in serum was determined by a time-resolved immunofluorometric assay as earlier described (Alfthan *et al*, 1988). With a sample volume of 25 μl in a total assay volume of 225 μl the detection limit was 0.45 pmol l^−1^. The upper reference limit of the assay for hCGβ in serum was 2 pmol l^−1^. The reference range is identical in women and men and it is not dependent on age. Serum creatinine was measured by a routine method in the Laboratory of Clinical Chemistry, University Hospital, Umeå, Sweden. The upper reference limit was 125 μmol l^−1^.

### Statistical analysis

Differences in serum hCGβ concentrations in patients with various stages and grades and controls were analyzed by the Mann-Whitney *U*-test. Survival curves were plotted using the Kaplan-Meier method, and comparison of survival times was performed with the log-rank test. Serum hCGβ was also studied as a categorical variable using quartiles (hCGβ <0.8 pmol l^−1^, 0.8–1.2 pmol l^−1^, 1.2–1.95 pmol l^−1^ and hCGβ >1.95 pmol l^−1^). The independence of hCGβ as a predictor of survival was analyzed by multivariate analysis using the Cox proportional hazard model with serum hCGβ, nuclear grade and stage as input variables. The statistical endpoint was survival measured from the date of nephrectomy to date of death or date of last follow-up. Cases of death from unrelated causes were censored. All tests were two-sided and *P*-values below 0.05 were considered significant.

## RESULTS

The median concentration of creatinine in serum of RCC patients was 86 μmol l^−1^ (range 45–353 μmol l^−1^), and elevated levels occurred in 13%. The serum concentrations of hCGβ were not related to serum creatinine.

The concentration of hCGβ in serum was elevated (>2 pmol l^−1^) in 23% of the patients with RCC (Table 2

**Table 2:**
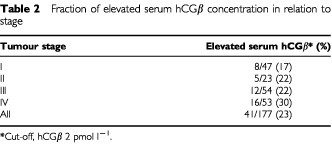
Fraction of elevated serum hCGβ concentration in relation to stage

) and 20 of these (11%) had values >4 pmol l^−1^. The median concentration of hCGβ in serum was 1.2 pmol l^−1^ (range 0.2–18 pmol l^−1^), which was significantly higher (*P*<0.0001) than in controls (median 0.4 pmol l^−1^, range 0.2–1.3 pmol l^−1^). There was no difference in hCGβ levels between males and females, different age groups, different RCC types, aneuploid and diploid tumours, or tumours with and without venous invasion. Serum hCGβ concentrations were not either significantly correlated with tumour stage or grade (Figure 1

**Figure 1:**
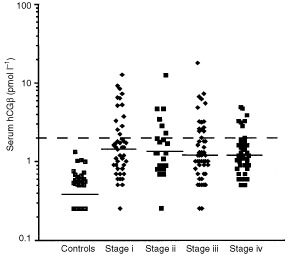
The distribution of hCGβ serum concentrations in controls and patients with various stages of RCC. The dashed line indicates the upper reference limit.

).

**Figure 2:**
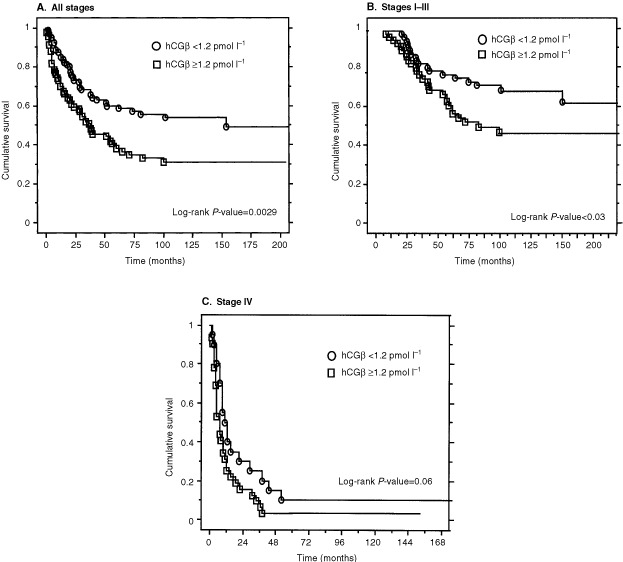
Kaplan Meier cancer specific survival according to preoperative serum hCGβ concentration in patients with RCC. Results for patients with tumours of all stages (**A**), stages I-III (**B**) and stage IV (**C**). The median value of the patients (1.2 pmol l^−1^) was used as a cut off.

Clinical stage and grade were highly predictive of disease specific survival (*P*<0.0001 each) in univariate analysis. Patients with serum hCGβ concentrations above the median value (1.2 pmol l^−1^) had significantly shorter survival than those with lower levels (*P*=0.0029) (Figure 2A

). A difference in survival time was observed also among patients with metastasized tumours (stage IV, Figure 2C) (*P*=0.06). When serum hCGβ concentration was compared as quartiles there was no difference in disease specific survival between the two lowest quartiles or between quartiles 3 and 4 (*P*>0.6). In multivariate analysis using the Cox regression model with age, gender, serum hCGβ, nuclear grade and stage as input variables, stage, grade and serum hCGβ concentration were independently associated with the disease specific survival (Table 3

**Table 3:**
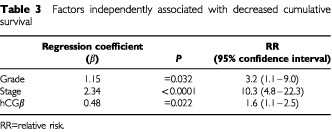
Factors independently associated with decreased cumulative survival

).

## DISCUSSION

RCC is known for its unpredictable clinical behaviour. Recurrence can occur many years after surgery and metastases can spontaneously regress after removal of the primary tumour. In addition to clinical stage and grade, DNA ploidy has been found to be a prognostic factor (Ljungberg *et al*, 1996), but a marker available prior to surgery could be used to optimize treatment. Of the many serum markers studied, only a few, i.e. VEGF, interleukin-10, CA-125, and TATI are of potential prognostic value (Grankvist *et al*, 1997; Jacobsen *et al*, 2000; Paju *et al*, 2001; Wittke *et al*, 1999). Clinical parameters such as performance status, serum lactate dehydrogenase, hemoglobin, serum calcium and prior nephrectomy have also been used to predict survival of patients with metastatic RCC (Motzer *et al*, 1999).

HCG immunoreactivity occurs in many different molecular forms in serum and urine. During pregnancy, intact hCG is the main form in serum and the proportion of hCGβ is 1–8%. Various forms of hCG can also be detected in serum of non-pregnant women and men by highly sensitive methods. The concentrations of hCG in serum and hCG and hCGβ in urine increase with age, whereas those of hCGβ in serum are similar in men and women and change very little with age (Alfthan *et al*, 1992b). In addition to being a sensitive marker for trophoblastic tumours of placental or gonadal origin, hCGβ is also expressed by many non-trophoblastic tumours including ovarian, gastrointestinal and squamous cell carcinomas. Contrary to trophoblastic tumours, these very seldom express intact hCG. A very moderate (up to two-fold) increase of hCGβ in serum may occur in patients with benign pancreatic and hepatobiliary diseases, but this does not invalidate the use of hCGβ as a tumour marker (Alfthan *et al*, 1992a).

Expression of hCGβ has been observed in various tumours and cell lines on both at the protein and mRNA level (Alfthan *et al*, 1992a; Iles *et al*, 1989; Ind *et al*, 1997; Sheaff *et al*, 1996), but there is little data on the expression in renal tumours. Increased concentrations of hCGβ have been demonstrated by radioimmunoassay in concentrated urine specimens from RCC patients (Fukutani *et al*, 1983), but the study included very few cases. The present study shows that the concentrations of hCGβ in serum were elevated in 23% of the patients, and the median concentrations were significantly higher than in controls (*P*<0.0001). Patients with serum hCGβ levels above the median value (1.2 pmol l^−1^) had significantly shorter survival than those with lower levels (*P*<0.0029). In multivariate analysis serum hCGβ, tumour stage and grade were independent prognostic variables. The fairly low frequency (23%) of elevated serum hCGβ suggest that this marker is expressed by only a part of the RCCs. However, because normal levels between 1.2 and 2 pmol l^−1^ were associated with adverse prognosis, it is possible that part of the hCGβ in these patients is derived from the tumour. A possible correlation with the response to treatment could not be evaluated in this study. The absence of a correlation with stage indicates that elevated levels are not only a result of tumour burden, but hCGβ expression rather characterizes a subgroup of tumours. The lack of significant correlation with established prognostic variables such as grade, stage and DNA-ploidy demonstrates the independent character of this marker.

HCGβ expression has been regarded as a characteristic of malignant transformation or dedifferentiation of cells, and its association with aggressive disease in transitional cell carcinoma of the bladder is well documented (Iles *et al*, 1989). The association with poor prognosis in RCC is in line with the results for other tumours. Free hCGβ has no known hormonal activity, but it has been shown to stimulate phospholipid methylation in rat Leydig cells (Ronco *et al*, 1993), rendering it a potential biological role. The three dimensional structure of hCGβ resembles that of the cysteine knot growth factors (Lapthorn *et al*, 1994), and it has a stimulating effect on the growth of cancer cells *in vitro*, suggesting paracrine or autocrine growth factor-like activity. Recently hCGβ has been shown to confer immortality features to cells by inhibiting apoptosis (Butler *et al*, 2000). This is a potential explanation for aggressive tumour behaviour of hCGβ expressing tumours.

The clinical utility of hCGβ in the management of RCC is limited by the infrequent expression. About one fourth of the patients had elevated levels and in half of these the elevation was substantial. However, a prognostic value was observed even with normal levels exceeding the median value, and this was the case also in patients with advanced disease. Thus it appears worthwhile to study whether hCGβ could be used as an aid in the selection of therapy or in monitoring of the disease after primary therapy. In conclusion, our results indicate that hCGβ in serum is a prognostic factor independent of stage and grade that can be used to identify a subgroup of patients with increased risk of aggressive disease.
